# Targeting Emerging Pathogenic Mechanisms by Natural Molecules as Potential Therapeutics for Neurodegenerative Diseases

**DOI:** 10.3390/pharmaceutics14112287

**Published:** 2022-10-25

**Authors:** Yan Dou, Dongju Zhao

**Affiliations:** 1 Department of Radiology and Tianjin Key Laboratory of Functional Imaging, Tianjin Medical University General Hospital, Tianjin 300052, China; 2 School of Life Sciences, Tianjin University, Tianjin 300072, China

**Keywords:** neurodegenerative diseases, natural molecules, ferroptosis, energy metabolism disorders, autophagy-lysosomal dysfunction, endoplasmic reticulum stress, gut dysbiosis, intracerebral delivery strategy

## Abstract

Natural molecules with favorable safety profile and broad pharmacological activities have shown great promise in the treatment of various neurodegenerative diseases (NDDs). Current studies applying natural molecules against NDDs mainly focus on well-recognized conventional pathogenesis, such as toxic protein aggregation, oxidative stress, and neuroinflammation. However, accumulating evidence reveals that some underlying pathogenic mechanisms are involved earlier and more deeply in the occurrence and development of NDDs, such as ferroptosis, energy metabolism disorders, autophagy-lysosomal dysfunction, endoplasmic reticulum stress, and gut dysbiosis. Therefore, determining whether natural molecules can play therapeutic roles in these emerging pathogenic mechanisms will help clarify the actual targets of natural molecules and their future clinical translation. Furthermore, how to overcome the inability of most poorly water-soluble natural molecules to cross the blood–brain barrier is also critical for effective NDD treatment. This review summarizes emerging pathogenic mechanisms targeted by natural molecules for NDD treatment, proposes nanocarrier-based drug delivery and intranasal administration to enhance the intracerebral bioavailability of natural molecules, and summarizes the current state of clinical research on natural product-based therapeutics.

## 1. Introduction

Neurodegenerative diseases (NDDs), including Alzheimer’s disease (AD), Parkinson’s disease (PD), Huntington’s disease (HD), and Amyotrophic Lateral Sclerosis (ALS), are characterized by progressive degradation of neuronal structure and function in the central nervous system [1]. Among them, AD is the most common NDD with cognitive impairment as the main clinical performance, and its main pathological features are deposition of amyloid-β (Aβ) protein and neurofibrillary tangles composed of hyperphosphorylated tau protein. PD presents with movement disorders such as bradykinesia, tremor, and unsteady gait, commonly attributed to the loss of nigrostriatal dopaminergic neurons and aggregates of α-synuclein. HD is a devastating inherited NDD caused by the mutant huntingtin (mHTT) gene that induces non-functional or toxic misfolded proteins, characterized by choreiform movements, psychiatric abnormalities, and cognitive deficits. ALS is known for rapid progression of severe muscle weakness and atrophy owing to the degeneration of motor neurons in brain and spinal cord, usually leading to death due to respiratory failure within two to five years of onset.

Although the clinical symptoms and pathological manifestations of NDDs are well recognized, they are still incurable. The incidence of NDDs not only rises rapidly with the aging of the world’s population, but also presents a younger trend in recent years. Existing drugs can only relieve some symptoms but fail to block disease progression, so there is an urgent need to explore effective therapeutic strategies to combat NDDs. Given that the pathogenesis of NDDs is complex and still not fully elucidated, there is a prevailing view that the most widely studied pathological manifestations such as characteristic protein aggregation are only the consequences rather than the causes of disease development. Some conventional pathogenic mechanisms, such as oxidative stress, neuroinflammation, and mitochondrial dysfunction, have been recognized to be closely related to the progression of NDDs. Specifically, oxidative stress caused by excessive production and weak clearance of reactive oxygen species (ROS) and reactive nitrogen species can lead to the oxidation of intracellular biomolecules for neuronal apoptosis. Aberrant activated glial cells can secrete large amounts of pro-inflammatory cytokines, inducing chronic neuroinflammation to damage neuronal structure and function. Abnormal mitochondrial division, fusion, and degradation can alter energy production and signal transduction in neurons or glial cells, resulting in neuronal death and synaptic loss. In contrast, several emerging pathogenic mechanisms have recently been discovered to be involved earlier and more deeply in the occurrence and development of NDDs, including ferroptosis, energy metabolism disorders, autophagy-lysosomal dysfunction, endoplasmic reticulum (ER) stress, and gut microbiota dysbiosis [2,3,4,5,6].

Among the numerous potential drugs, natural molecules mainly derived from plants show excellent biosafety and various beneficial pharmacological activities [7]. Many studies have revealed that natural molecules are effective against a variety of intractable diseases, such as cardiovascular disease, cancer, and NDDs [8,9,10,11,12]. At present, the potential targets of neuroprotective effects of natural molecules are mainly focused on well-recognized conventional pathogenesis, such as toxic protein aggregation, oxidative stress, and neuroinflammation [10,13]. For example, berberine and curcumin have been investigated to inhibit Aβ and α-synuclein accumulation in AD model APP/tau/PS1 transgenic mice and lipopolysaccharide-induced PD mouse model, respectively [14,15]. 3-alkyl luteolin derivatives were found to decrease oxidative stress in HD mouse striatal cells [16], and Ginsenoside Re could attenuate neuroinflammation in a symptomatic human-superoxide dismutase 1 (hSOD1^G93A^) mouse model of ALS [17]. However, due to the wide range of pharmacological effects of natural molecules, it is of great significance to determine whether natural molecules can play therapeutic roles in these above-mentioned emerging pathogenic mechanisms, which will contribute to clarifying the actual targets of natural molecules and their future clinical translation.

This review highlights several popular and fascinating emerging pathogenic mechanisms as potential therapeutic targets of current natural molecules for NDD treatment. Subsequently, the alternative administration route to improve the intracerebral bioavailability of natural molecules and related clinical trials in progress are discussed.

## 2. Emerging Pathogenic Mechanisms Targeted by Natural Molecules

**Figure 1:**
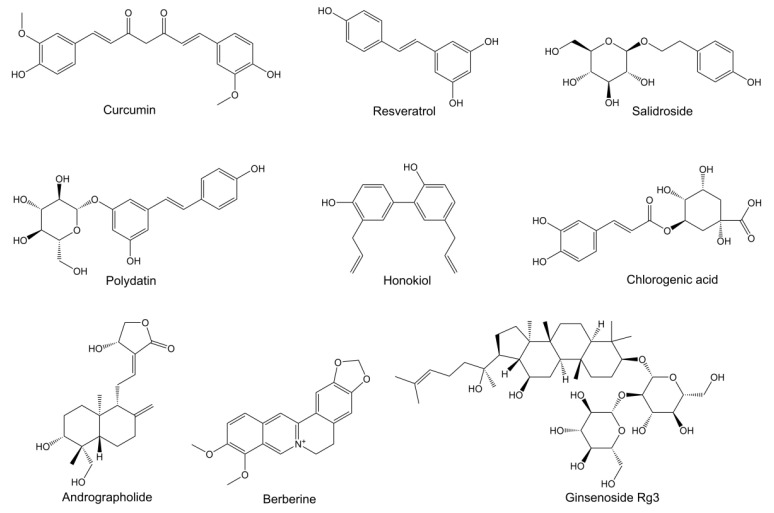
Chemical structures of the main natural molecules examined.

This section evaluates the momentous roles of emerging pathogenic mechanisms targeted by natural molecules in NDDs, including ferroptosis, energy metabolism disorders, autophagy-lysosomal dysfunction, ER stress, and gut microbiota dysbiosis. These natural molecules refer to single-molecule bioactive natural products with specific chemical structures, among which the structural formulas of the most studied natural molecules targeting these emerging pathogenic mechanisms are shown in Figure 1. The specific emerging pathogenic mechanism corresponding to each subheading and the evidence of targeted regulation of natural molecules are detailed as follows. Firstly, each pathogenic mechanism and its impact on NDDs progression are elaborated to reveal the importance of targeting these emerging mechanisms for the treatment of NDDs. Secondly, several natural molecules are proposed as representative examples to show their modulations for specific pathogenic mechanisms through different pathways. Currently reported natural molecules, their specific regulatory effects, and corresponding references are comprehensively summarized in the following tables, providing powerful evidence for natural molecules targeting these underlying pathologies. Finally, the current status and limitations of the research are critically discussed to put forward potential research directions.

### 2.1. Ferroptosis

Ferroptosis is a novel iron-dependent cell death driven by the iron overload and lipid peroxidation, which is distinct from the programmed cell death [2]. Smaller mitochondria, thicker membrane density, and excessive ROS are typical features of ferroptotic cells. A series of proteins, represented by transferrin, ferritin, and ferroportin 1, are responsible for iron uptake, storage, and export, respectively, to maintain intracellular iron homeostasis. Aberrant expression of these proteins leads to an imbalance in iron metabolism and iron overload, which have been found in the brains in the early stages of some NDDs [2]. These superabundant iron radicals generate large amounts of ROS through the Fenton reaction, which can disrupt the fragile bis-allyl chemical bonds of polyunsaturated fatty acids (PUFAs) in membrane components. Subsequent oxidative cascades lead to massive accumulation of toxic lipid peroxide molecules, which eventually destroy the structure and biological function of cellular and organelle membranes to induce neuronal death. Furthermore, the body’s own antioxidant responses are also reduced in the pathological process of NDDs [18]. For example, antioxidant signaling pathways such as the nuclear factor erythroid 2-related factor 2 (Nrf2) are inhibited, resulting in the downregulation of various downstream antioxidant enzymes, such as glutathione peroxidase 4 (GPX4) and heme oxygenase-1 (HO1), further aggravating ferroptosis-induced oxidative damage and neuronal loss.

Several natural molecules have been reported to inhibit ferroptosis for NDD treatment. For example, curcumin has been shown by Du et al. to possibly exert iron-chelating activity to reverse excess iron-induced dopamine depletion and nigral dopaminergic neuronal degeneration in the brain of 6-hydroxydopamine (6-OHDA)-induced PD model rats [19]. Ginkgolide B, a terpene lactone derivative of *Ginkgo biloba*, has been revealed by Shao et al. to ameliorate AD-related cognitive impairment in senescence-accelerated P8 (SAMP8) mice, through reducing iron content in the brain, decreasing transferrin receptor 1 (TFR1) and nuclear receptor coactivator 4 (NCOA4) expressions, increasing ferritin heavy chain (FTH1) expression, and activating the Nrf2/GPX4 signaling pathway [20]. Salidroside, a precious phenylethanoid glycoside derived from *Rhodiola*, has been found by Yang et al. to effectively decrease the levels of intracellular Fe^2+^ to attenuate lipid peroxidation and mitochondrial damage in glutamate-induced HT22 cells, while increasing expressions of GPX4 and solute carrier family 7 membrane 11 (SLC7A11) and activating the Nrf2/HO1 signaling pathway in Aβ_1-42_-induced AD mice [21].

Besides representative compounds discussed above, more natural molecules reported to inhibit ferroptosis are summarized in Table 1. Overall, several natural molecules against ferroptosis have been investigated to combat AD and PD in vitro and in vivo. However, whether natural molecules can treat HD and ALS by inhibiting ferroptosis is unclear and needs more exploration in the future. The inhibitory effect of natural molecules on ferroptosis is mainly dependent on their iron-chelating properties, so it is necessary to discuss the difference of different natural molecules in reducing iron content in order to select the natural molecules with the best performance. Furthermore, assessment of subsequent pathologies such as lipid peroxidation caused by iron overload, as well as functional improvements in the body’s own iron exclusion pathways, are also important to fully determine the inhibitory effects of natural molecules on ferroptosis.

### 2.2. Energy Metabolism Disorders

Cells require enough energy to sustain the body’s normal life activities. Despite accounting for only 2% of body weight, the brain consumes 20% of the body’s total energy, showing a high demand for energy in the brain [3]. Glucose is the main energy source in the brain and is transported from the blood to the brain parenchyma through the coordination of glucose transporters on capillary endothelial cells and astrocytes. Concretely, glucose is transported to astrocytes mediated by glucose transporter type 1 (GLUT1) in vascular endothelial cells, and then to neurons mediated by GLUT3 and GLUT4 in astrocytes. Pathological factor-induced astrocyte degeneration in NDDs leads to abnormal glucose transport, thereby disrupting normal energy metabolism in the brain. After being taken up by neurons, glucose is converted into pyruvate in the cytoplasm and then into acetyl coenzyme A in the mitochondria to generate ATP via the tricarboxylic acid cycle, which directly provides energy for neuronal activity. Decreased enzymatic activity and mitochondrial dysfunction in NDDs can halt ATP production.

Previous studies have demonstrated that reduced glucose utilization and ATP production are found in the brains in early stages of AD, PD, HD, and ALS [24]. Furthermore, lactate and lipid metabolism are also important components of energy metabolism. Decreased brain glucose uptake in NDDs results in an increased proportion of anaerobic glycolytic metabolism, resulting in the inability of pyruvate to enter the mitochondria but being converted into large amounts of lactate. However, how lactate homeostasis contributes to the pathology of NDD remains inconclusive. Lipids have been identified as energy reservoirs in the brain, including phospholipids, sphingolipids, glycerolipids, fatty acids, and sterols, which are essential for maintaining the normal structure and function of neurons and myelin sheaths [25]. Lipid metabolism disorders lead to abnormal signal transduction, impair ion channels and synaptic plasticity, and promote neuronal death, thereby inducing the occurrence and development of NDDs [26,27].

Some natural molecules have shown neuroprotective effects by improving energy metabolism disorders in the brain. For example, Wang et al. found that curcumin could enhance brain glucose uptake in APP/PS1 mice, a common AD model, by upregulating the protein expression of GLUT1 and GLUT3 [28]. Li et al. revealed that honokiol might enhance energy metabolism in AD PS1_V97L_ transgenic mice by increasing mitochondrial SIRT3 expression and enhancing ATP production [29]. Zhang et al. investigated the neuroprotective effects of polydatin on early dopaminergic neuronal degeneration in 1-methyl-4-phenyl-1,2,3,6-tetrahydropyridine (MPTP)-induced PD mice, by inhibiting glycolysis, increasing pyruvate content, and enhancing ATP production [30]. Lu et al. found that curcumin enhanced lactate content in the brains of APP/PS1 mice to provide energy and improve cognitive function [31]. However, Wang et al. found that administration of lactate to wild-type mice disrupted hippocampal neurogenesis, while reducing lactate levels in phosphatase and tensin homolog (PTEN) floxed mice restored hippocampal neurogenesis and cognitive function [32]. Therefore, the improvement of lactate energy supply by natural molecules in NDDs needs further studies.

Table 2 summarizes the detailed therapeutic mechanisms of current natural molecules against energy metabolism disorders, with emphasis on in vivo studies. Existing studies are still preliminary exploration and lack of in-depth mechanism research. Firstly, since there are different types of brain cells involved in energy metabolism, such as astrocytes, microglia, and neurons, studying the specific effects of natural molecules on energy metabolism in different cell types can help identify precise targets for natural molecules. Secondly, energy metabolism involves a variety of biological processes, including different metabolic pathways of glucose, lactate, and lipid metabolism; thus, distinguishing which process is the main pathway affected by natural molecules is also a potential research direction in the future. Finally, it is important to identify the gene targets of natural molecules that affect energy metabolism to analyze the specific regulatory pathways.

### 2.3. Autophagy-Lysosomal Dysfunction

Autophagy-lysosomal pathway or autophagy is an important self-protective biological mechanism that sequesters toxic protein aggregates and damaged organelles, playing a vital role in cellular homeostasis. These undesirable substances are phagocytosed into the cell-derived double member structure called autophagosomes, which subsequently fuse with lysosomes and are degraded by various hydrolases into amino acids and fatty acids for cellular energy [4]. However, under pathological conditions of NDDs, the disruption of autophagosome-lysosome fusion and lysosomal dysfunction fail to eliminate toxic contents, leading to the occurrence and pathological deterioration of NDDs. A recent review revealed that autophagy was the main route to remove abnormally toxic Aβ and tau proteins in AD pathogenesis. In addition, mounting evidence from post-mortem analysis in AD patients and AD animal models illustrated impairment of the autophagy-lysosomal pathway in AD [36]. Similarly, α-synuclein, the key pathologicalhallmark of PD, was degraded by autophagy, and abnormal expression levels for the genes encoding autophagy-related proteins have been identified by some evidence in PD [37]. Some other researchers described the findings for the role of autophagy in eliminating misfolded neurotoxic proteins for HD and ALS [38,39].

Natural molecules have been investigated as autophagic inducers for NDD treatment. For example, berberine, derived from the roots and bark of *Coptis chinensis*, has been usually used to treat infectious diseases such as gastroenteritis, and recent studies have shown its promising therapeutic effects in HD, AD, and PD models. Jiang et al. found that berberine could decrease mHTT aggregates both in green fluorescent protein (GFP)-exon1 HTT containing transfected HEK293 cells and transgenic N171-82Q mice as HD models via activating the autophagy-lysosomal pathway [40]. Berberine was also found by Huang et al. to promote Aβ and tau clearance by enhancing autophagic activity in the hippocampus of 3 × Tg AD mice [41,42]. Furthermore, as reported by Deng et al., berberine reduced the level of α-synuclein and enhanced AMP-activated protein kinase (AMPK) dependent-autophagy in PD cell and mouse models [43]. Mancuso et al. revealed that resveratrol treatment promoted motoneuron survival and improved locomotion impairment in the SOD1^G93A^ ALS mouse model by enhancing sirtuin 1 (SIRT1)/AMPK-related autophagic flux [44].

Table 3 summarizes the current natural molecules targeting the autophagy-lysosomal pathway and their detailed therapeutic mechanisms. It is noteworthy that natural molecules promote the elimination of toxic proteins mainly through the autophagy-lysosomal pathway against NDDs. Therefore, it would be of further interest to investigate how natural molecules affect the process of directing the transport of these toxic proteins to autophagic lysosomes. In addition, identifying the gene targets and signaling pathways involved in the autophagy-lysosomal pathway is the key to evaluating the efficacy of natural molecules. Since autophagy has a double-edged sword effect, it is critical to determine the optimal stage for natural molecules to promote autophagy and maintain normal homeostasis to optimize the efficacy of natural molecules for NDDs treatment. To further clarify the therapeutic effects of natural molecules to promote autophagy, it may be necessary to compare with classic commercialized autophagy activators such as rapamycin in future fundamental and clinical experiments.

### 2.4. Endoplasmatic Reticulum Stress

ER stress is an important defense mechanism of cells, maximizing resistance to internal and external stress. ER is an important organelle involved in the synthesis, processing, and transport of proteins and the storage of Ca^2+^. Under physiological conditions, misfolding and unfolded proteins in the ER lumen and Ca^2+^ imbalance can activate the unfolded protein response and promote the refolding or degradation of abnormal proteins [5]. Therefore, normal ER stress is conducive to maintaining cellular homeostasis, whereas persistent or intense ER stress can trigger programmed cell death or apoptosis in the progression of NDDs. There are two main pathways for ER stress-mediated apoptosis: C-EBP homologous protein (CHOP) and jun-N terminal kinase (JNK) signaling pathways [50]. CHOP is an ER stress-specific protein, and its expression increases with enhanced ER stress, which increases the expression levels of pro-apoptosis-related proteins and promotes Ca^2+^-mediated mitochondrial apoptosis to induce apoptosis. Similarly, JNK activation by ER stress induces the phosphorylation of B-cell lymphoma-2 (BCL-2) family members to increase mitochondrial apoptosis and upregulates Beclin1 expression to cause autophagy-dependent cell death.

Previous studies have reported some natural molecules targeting ER stress against NDDs. For instance, echinacoside isolated from *Cistanche tubulosa* exhibits efficient neuroprotective effects in multiple ways, such as ameliorating mitochondrial dysfunction, scavenging ROS, and restraining neuroinflammation to treat NDDs [51]. Zhang et al. demonstrated that echinacoside protected dopaminergic neurons from ER stress-induced death by promoting seipin degradation and the GRP94/BIP-ATF4 (glucose-regulated protein 94/binding immunoglobulin protein-activating transcription factor 4)-CHOP signalling pathway in a 6-OHDA-induced PD model [52]. In a recent study by Dai et al., echinacoside treatment effectively inhibited ER stress in hippocampal neurons of APP/PS1 mice through activating the PERK/eIF2α (protein kinase RNA-like endoplasmic reticulum kinase/eukaryotic translation initiation factor-2α) signalling pathway, indicating that ER stress is a key therapeutic target of echinacoside for AD treatment [53].

The natural molecules that have been currently reported to inhibit ER stress against NDDs are summarized in Table 4. Most of the current studies have focused on the effect of natural molecules in reducing ER stress, but not much attention has been paid to the degradation and clearance of misfolded proteins caused by ER stress. Therefore, it is necessary to further investigate whether natural molecules can activate ubiquitination and proteasome degradation pathways or autophagy-lysosomal pathways while weakening ER stress. Since energy metabolism disorder is one of the causes of ER stress, and ER stress is closely associated with oxidative stress and neuroinflammation, it is important to consider the interrelationship between different pathologies when formulating effective treatment strategies targeting ER stress for NDD treatment. In addition, how to ensure that the application of natural molecules does not excessively weaken ER stress and make it still play its normal defense function is also worth exploring.

### 2.5. Gut Dysbiosis

In recent years, the role of gut microbiota in the central nervous system has attracted increasing attention. The intestinal microbes are mainly composed of pathogenic and beneficial bacteria, which are in a dynamic balance to maintain host homeostasis in normal physiological states [63]. Gut microbiota can establish a bidirectional communication with the brain through immune, metabolic, and neural pathways, known as the microbiota–gut–brain axis. Some sustained risk factors, such as genetic variation, aging, negative emotions, and improper diet, can alter the relative species abundance of gut microbiota, leading to gut dysbiosis [64]. Clinical studies have shown that gut dysbiosis always appears before neurological symptoms and plays a non-negligible role in the occurrence and development of NDDs [6]. Firstly, gut dysbiosis leads to persistent activation of immune cells in the enteric nervous system and peripheral blood, thereby activating immune signalling pathways and inducing chronic neuroinflammation in the central nervous system. Secondly, gut dysbiosis disrupts the integrity of the intestinal barrier, leading to the entry of pathogenic bacteria and toxic metabolites into the peripheral systemic circulation and then into the central nervous system, aggravating NDD pathologies. Thirdly, various risk stimuli affect the normal secretion of neurotransmitters by enteric neurons and enterocytes, hinder their afferents to the brain through the vagus nerve, and block signal transmission to the central nervous system.

Current studies show that natural molecules can restore the gut microbiota composition to target gut dysbiosis in NDDs. Silymarin, the extract of milk thistle, has been manufactured as a commercial agent to combat liver disorders, such as alcoholic liver diseases, viral hepatitis, and cirrhosis. Shen et al. found that silymarin and its major active compound, silybin, alleviated memory deficits and reduced amyloid plaque burden in APP/PS1 mice, which may be attributed to regulate gut microbiota composition and microbiota diversity decline [65]. A recent study by Xie et al. explored the neuroprotective effects and mechanisms of salidroside in SAMP8 mice as an AD model, and found that it not only improved the gut barrier integrity and modified the gut microbiota, but also decreased the levels of proinflammatory cytokines in the peripheral circulation and the central nervous system [66].

Table 5 lists alternative natural molecules for ameliorating gut dysbiosis, mainly for AD treatment. Although several preclinical and clinical trails have demonstrated the association between gut dysbiosis and pathological changes in NDDs, natural molecules targeting gut dysbiosis to intervene in NDDs are seldom reported and need to be further explored. Interestingly, ellagic acid, a natural polyphenolic compound, as well as its gut microbiota metabolites’ urolithins have been found to regulate gut microbiota, which has not been investigated in NDD treatment [67,68]. Furthermore, most current studies on ameliorating gut dysbiosis have focused on regulating the projections of gastrointestinal signals to the central nervous system, while, in turn, the effects of toxic proteins or inflammatory factors in the brain during NDDs progression on gut microbiota are poorly studied, which is also an extremely valuable research direction for thorough and accurate evaluation of the brain–gut axis.

## 3. Intracerebral Administration Strategies for Natural Molecules

Although natural molecules in the existing studies were all tested in vivo by gavage and intraperitoneal injection for in vivo experiments (Table 1, Table 2, Table 3, Table 4 and Table 5), enhancing their bioavailability and efficiency into the brain remains challenging. The blood–brain barrier (BBB) protects the brain from exogenous toxic stimulation, but also prevents most poorly water-soluble natural molecules from entering the brain parenchyma. Therefore, enhancing the intracerebral bioavailability of natural molecules is beneficial to improve their efficacy against NDDs.

Nanotechnology-based drug carriers can not only improve the solubility and biocompatibility of natural molecules through entrapping, but also mediate the crossing of the BBB through surface-modified ligands, which have emerged as an effective alternative strategy for the delivery of natural molecules into the brain. A current study by Qu et al. showed that the relative bioavailability of nano-honokiol by gavage was significantly increased by 1.9-fold than that of free honokiol through improving its solubility, thereby better inhibiting neuropathology and modulating gut microbiota to alleviate cognitive impairment in TgCRND8 mice for AD treatment [74]. Li et al. designed resveratrol-based nanoparticles modified with the BBB transport-targeting peptide TGN (TGN-Res@SeNPs) for AD treatment, and their in vitro BBB transport efficiency was 75%, much higher than the 12% of the unmodified nanoparticles (Res@SeNPs) and 4% of free resveratrol [75]. Liu et al. used focused ultrasound and microbubbles to open the BBB to deliver quercetin for reducing ER stress-induced AD pathology [76]. Although natural molecule-based nanoformulations targeting emerging pathogenic mechanisms have been shown to be effective in AD treatment, their therapeutic effects in PD, HD, and ALS need further exploration. In addition, inconsistent criteria for assessing nanomedicine quality and efficacy are a major obstacle and challenge for natural molecule-based nanoformulations’ development.

Intranasal administration has become a promising strategy to deliver drugs into the brain via bypassing the BBB [77]. Therapeutic agents are directly transported from the nose to the brain through olfactory and trigeminal nerve pathways, requiring shorter transportation time to enter the central nervous system than traditional administration routes into the systemic circulation. However, intranasal administration is usually suitable for a small volume administration of highly concentrated water-soluble drugs, while natural molecules are generally difficult to dissolve in water and have a limited ability to penetrate the nasal mucosa. Therefore, the therapeutic effect of using natural molecules alone via intranasal administration is not ideal enough [78]. Recently, intranasal administration in combination with nanoformulations is beneficial to fully exploit the advantages of intranasal administration. Our previous work reported a nanoformulation of quercetin entrapped by human serum albumin to exert excellent antioxidant therapeutic effects in 11-month-old APP/PS1 mice via intranasal administration [79]. Liu et al. developed a self-assembled curcumin analogue nanoformula (NanoCA) that was delivered into the brain by a rapid arousal intranasal delivery system for PD therapy [80]. In the future, more focus should be placed on the pharmacokinetics, metabolism, and distribution of natural molecule-based nanoagents administered intranasally, which are currently few but crucial for clarifying pharmacological effects and promoting clinical translation of natural molecules.

## 4. Clinical Trails of Natural Molecules for NDD Treatment

Given the above-described excellent therapeutic potential and the natural safety profile, natural molecules show excellent promise for clinical translation into NDD treatment. Therefore, it is necessary to conduct clinical trials to test the actual therapeutic effects of natural molecules in human NDD patients. Table 6 summarized natural molecule-based therapeutic agents currently in clinical trials for NDDs, screened by disease and drug as key items from the website (https://clinicaltrials.gov, accessed on 20 August 2022) of the Comprehensive Database of Clinical Trials administered by the U.S. Food and Drug Administration (FDA) or the National Institutes of Health. Most of the listed clinical trials have been approved for execution within the last 3–5 years, and less than half have just begun recruiting subjects. The goal of these clinical reagents is to demonstrate the safety and efficacy of natural molecule-based therapeutics, with little focus on specific mechanisms of action or only for conventional pathogenesis and lack of in-depth exploration of emerging pathogenic mechanisms. Among the current clinical trials targeting the four most common NDDs, AD is the most widely investigated disease treated by natural molecules, while the clinical research of natural molecules for the treatment of other NDDs needs to be further explored and carried out. In addition, clinical trials of natural molecule-based nanomedicines are also highly anticipated in the future, but there are still many obstacles and more preclinical research support is needed.

## 5. Conclusions

An increasing number of studies have revealed that several emerging pathogenic mechanisms play significant roles in the occurrence and development of NDDs, and they are beginning to be explored as efficient therapeutic targets for natural molecules. The rapidly increasing incidence and younger trend of NDDs have prompted researchers to pay attention to the early and upstream pathogenesis to develop effective drugs for NDD prevention and treatment. Undoubtedly, natural molecules are a competitive therapeutic option, which not only have the advantages of natural safety unmatched by other chemical drugs, but also exhibit a good inhibitory effect on the emerging early pathogenesis. Nanocarrier-based drug delivery technology can enhance the intracerebral bioavailability of natural molecules that are inherently difficult to cross the BBB. Meanwhile, intranasal administration with high patient compliance can facilitate rapid brain entry of natural molecules that are not limited by the BBB. These two strategies are helpful to improve the efficacy and clinical translation of natural molecules for NDDs. In addition, clinical trials evaluating the efficacy of natural molecules for NDDs are currently underway, and further clinical studies are needed to clarify the specific therapeutic mechanisms. Taken together, based on the therapeutic potential of natural molecules, it is essential to separately and deeply evaluate their chemical composition, mechanisms of action, therapeutic repeatability, and human applicability, which are prerequisite for future translation.

## Figures and Tables

**Table 1:** Natural molecules inhibit ferroptosis for NDD treatment.

Compounds	Models	Mechanisms	Administration	Ref.
Curcumin	6-OHDA induced PD rats	Suppressing the iron-induced degeneration of nigral dopaminergic neurons by iron-chelating activity	i.g.	[19]
7-O-Esters of taxifolin	Glutamate-induced HT22 cells; Aβ25-35-induced AD mice	Resisting oxytosis, ferroptosis and ATP depletion	i.p.	[22]
Ginkgolide B	SAMP8 mice as AD model	Mitigating ferroptosis by reducing iron content, decreasing TFR1 and NCOA4 expressions, increasing FTH1 expression, and activating the Nrf2/GPX4 signaling pathway	i.g.	[20]
Myricetin	Fe^2+^-induced SH-SY5Y cells; scopolamine-induced AD mice	Downregulating acetylcholinesterase and brain iron content; inhibiting TFR1 expression; increasing antioxidant enzyme activity	i.g.	[23]
Salidroside	Glutamate-induced HT22 cells; Aβ1-42-induced AD mice	Reducing lipid peroxidation and ROS levels; increasing GPX4 and SLC7A11 protein expressions; improving mitochondrial ultrastructure; attenuating neuronal ferroptosis by activating the Nrf2/HO1 signaling pathway	i.g.	[21]

ATP: adenosine triphosphate; TFR1: the receptor of transferrin to control cellular iron uptake. NCOA4 can mediate autophagic degradation of ferritin to maintain iron homeostasis; SLC7A11 can regulate GSH production; i.g. for intragastric administration; i.p. for intraperitoneal injection.

**Table 2:** Natural molecules improve energy metabolism disorders for NDD treatment.

Compounds	Models	Mechanisms	Administration	Ref.
Acteoside	Streptozotocin- induced AD mice	Increasing the protein expression of glucose transporters and glucose levels; enhancing ATP production; reducing the ROS level	i.g.	[33]
Andrographolide	J20 Tg mice as AD model	Increasing the uptake and utilization of glucose; enhancing ATP production	i.p.	[34]
Curcumin	APP/PS1 mice as AD model	Improving the glucose uptake in cerebrum; increasing the protein expression of GLUT1 and GLUT3; enhancing lactate content	i.g./i.p.	[28,31]
Ginsenoside Rg3	D-galactose-induced AD rats	Resisting the oxidative stress; regulating the energy metabolism-related biomarkers; recovering mitochondrial ETC perturbations	i.g.	[35]
Honokiol	Aβ1-42-induced primary hippocampal neurons; PS1_V97L_ Tg mice	Increasing mitochondrial SIRT3 expression; enhancing ATP production; weakening mitochondrial ROS production	i.p.	[29]
Polydatin	MPTP-induced PD mice	Inhibiting glycolytic metabolism; increasing pyruvate content; restoring ATP production level	i.g.	[30]

ETC: electron transport chain; SIRT3, sirtuin 3, a nicotinamide adenine dinucleotide-dependent deacetylase; i.g. for intragastric administration; i.p. for intraperitoneal injection.

**Table 3:** Natural molecules alleviate autophagy-lysosomal dysfunction for NDD treatment.

Compounds	Models	Mechanisms	Administration	Ref.
Astersaponin I	MPP^+^-induced SH-SY5Y cells; MPTP-induced PD mice	Upregulating autophagy by activating the ERK/mTOR and AMPK/mTOR pathways	i.g.	[45]
Berberine	GFP-exon1 HTT containing transfected HEK293 cells; transgenic N171-82Q mice as HD model	Promoting the degradation of mHTT by enhancing autophagic function	i.g.	[40]
	3 × Tg AD mice	Reducing Aβ production; facilitating Aβ clearance in an autophagy-dependent manner	oral	[41]
	3 × Tg AD mice	Reducing the accumulation of total tau and hyperphosporylated tau via the Akt/GSK3β pathway; promoting tau clearance by modulating the class III PI3K/Beclin-1 autophagic pathway	oral	[42]
	MPP+-induced SH-SY5Y cells; MPTP-induced PD mice	Enhancing autophagy and AMPK phosphorylation	i.g.	[43]
Chlorogenic Acid	Aβ25-35-induced SH-SY5Y cells; APP/PS1 mice as AD model	Enhancing autophagosome–lysosome fusion by decreasing autophagosome production and increasing autolysosome content; enhancing lysosomal activity via the mTOR/TFEB pathway	i.g.	[46]
Cinnamic acid	5xFAD mice	Activating the nuclear hormone receptor PPARα to transcriptionally upregulate TFEB; stimulating lysosomal biogenesis	i.g.	[47]
Curcumin analog C1	5xFAD mice, P301S mice, and 3xTg-AD mice	Activating TFEB; enhancing autophagy and lysosomal activity; reducing APP, APP CTF-β/α, Aβ peptide and tau aggregate content	oral	[48]
Ferulic Acid	Stable GFP-RFP-LC3 U87 cells and PC-12 cells; α-synuclein- and 6-OHDA-induced C. elegans as PD model	Inducing autophagy by inhibiting SQST-1/p62 protein expression and increasing mRNA levels of 3 key autophagy-related genes including lgg-1, vps-34, and unc-51	medium	[49]
Resveratrol	SOD1G93A ALS mice	Increasing expression and activation of SIRT1 and AMPK to promote normalization of the autophagic flux and increase mitochondrial biogenesis	oral	[44]

MPP+: 1-methyl-4-phenylpyridinium; ERK, extracellular regulated protein kinases; mTOR: mammalian target of rapamycin; Akt: protein kinase B; GSK3β: glycogen synthase kinase 3β; PI3K: phosphoinositide 3-kinase; TFEB: transcription factor EB; PPARα: peroxisome proliferator-activated receptor α; APP: β-amyloid precursor protein; CTF-β/α: C-terminal fragments; SQST-1:sequestosome related 1; SQST-1/p62, the autophagy substrate protein; i.g. for intragastric administration; i.p. for intraperitoneal injection; oral/medium for drugs mixed in the diet for mice or in the medium for *C. elegans.*

**Table 4:** Natural molecules mitigate excessive ER stress for NDD treatment.

Compounds	Models	Mechanisms	Administration	Ref.
Astragaloside-IV	MPP^+^-induced MN9D cells; MPTP-induced PD mice	Lessening the expression of CHOP protein by restraining the expression of lincRNA-p21	i.p.	[54]
Berberine	3 × Tg AD mice or APP/PS1 mice	Reducing Aβ production and ER stress by inhibiting PERK/eIF2α-mediated BACE1 translation	oral	[55,56]
Curcumin	ApoE4 transgenic mice as AD model	Inhibiting ER stress by reducing GRP78 and IRE1α expression	i.p.	[57]
Echinacoside	6-OHDA-induced PC12 cells and rats as PD model	Relieving ER stress by promoting seipin degradation and GRP94/BIP-ATF4-CHOP pathway; decreasing ROS accumulation	i.p.	[52]
	Aβ_1-__42_-induced SH-SY5Y cells; APP/PS1 mice as AD model	Reducing Aβ production and deposition by repressing BACE1 activity; inhibiting ER stress via the PERK/eIF2α pathway	i.g.	[53]
Epigallocatechin Gallate	Aβ_1-42_-induced SH-SY5Y cells; APP/PS1 mice as AD model	Mitigating ER abnormal ultrastructural swelling; downregulating GRP78, CHOP, cleaved-caspase-12 and -3 expressions	oral	[58]
Luteolin	APP23 mice as AD model	Preventing ER stress to suppress microglial activation by suppressing CHOP and IL-1β mRNA levels	i.p.	[59]
Quercetin	Okadaic acid-induced SH-SY5Y cells; high fat diet-induced AD mice	Suppressing ER stress by decreasing phosphorylation of IRE1α and PERK; inhibiting TXNIP and NLRP3 inflammasome activation; attenuating tau phosphorylation	oral	[60]
Resveratrol	Rotenone-induced PD rats	Ameliorating ER stress by downregulating CHOP and GRP78 expressions and hampering caspase-3 activity; restoring redox balance by suppressing xanthine oxidase activity and protein carbonyls formation and activating glutathione peroxidase and Nrf2 signaling pathway	i.g.	[61]
Tanshinone IIA	APP/PS1 mice as AD model	Preventing abnormal expression of GRP78, eIF2α, IRE1α, ATF6; suppressing the activation of CHOP and JNK pathways	i.p.	[62]

BACE1: β-site APP cleavage enzyme 1; IRE1α: inositol requiring enzyme-1α; TXNIP: thioredoxin-interacting protein; NLRP3: NOD-, LRR- and pyrin domain-containing protein 3; i.g. for intragastric administration; i.p. for intraperitoneal injection; oral for drugs mixed in the diet.

**Table 5:** Natural molecules ameliorate gut dysbiosis for NDD treatment.

Compounds	Models	Mechanisms	Administration	Ref.
Berberine	PD patients	Improving the disorder of intestinal flora and suppressing the expression of inflammatory factors	oral	[69]
Curcumin	APP/PS1 mice as AD model	Altering the relative abundances of bacterial taxa	i.g.	[70]
Gastrodin	D-galactose-induced AD mice	Changing the gut microbiome composition	i.g.	[71]
Ginkgolide B	D-galactose and aluminum chloride-induced AD mice	Reconstructing gut microbiota by reversing the decreased abundance of Lactobacillus and the increased abundance of Bacteroidales, Muribaculaceae, and Alloprevotella	i.g.	[72]
Ginsenoside Rg1	Aβ_25–35_-induced tree shrews as AD model	Changing the abundance of gut microbiota and increasing lactobacillaceae	i.g.	[73]
Salidroside	SAMP8 mice as AD model	Improving the gut barrier integrity and modifying the gut microbiota	i.g.	[66]
Silibinin and silymarin	APP/PS1 mice as AD model	Regulating the microbiota diversity and abundance of several key bacterial species associated with AD	i.g.	[65]

i.g. for intragastric administration, oral for drugs mixed in the diet.

**Table 6:** Natural molecule-based therapeutic agents currently in clinical trials for NDDs (available online: https://clinicaltrials.gov, accessed on 20 August 2022).

Natural Molecule-Based Agents	Disease	Status (CT.gov ID)	Phase	Date
Caffeine	AD	Recruiting (NCT04570085)	Phase 3	2021–2024
Colchicine	ALS	Active, not recruiting (NCT03693781)	Phase 2	2019–2022
Combination product: antioxidants	ALS	Recruiting (NCT04244630)	Phase 2	2022–2023
Conventional medication and chinese herbal medicine	PD	Not yet recruiting (NCT05001217)	Phase 2, 3	2021–2023
Curcumin and yoga	AD	Active, not recruiting (NCT01811381)	Phase 2	2014–2020
Dasatinib and quercetin	AD	Active, not recruiting (NCT04063124)	Phase 1, 2	2020–2022
Dasatinib and quercetin	AD	Enrolling by invitation (NCT04785300)	Phase 1,2	2022–2023
Dasatinib and quercetin	AD	Recruiting (NCT05422885)	Phase 1, 2	2022–2023
Dasatinib and quercetin	AD	Recruiting (NCT04685590)	Phase 2	2021–2032
DHA	AD	Active, not recruiting (NCT03613844)	Phase 2	2018–2025
Flos gossypii flavonoids tablet	AD	Recruiting (NCT05269173)	Phase 2	2020–2023
Ganoderma	PD	Recruiting (NCT03594656)	Phase 3	2018–2021
Huperzine A	AD	Not yet recruiting (NCT02931136)	Phase 4	2019–2025
Icosapent ethyl (IPE)	AD	Active, not recruiting (NCT02719327)	Phase 2, 3	2017–2023
Medical cannabis	PD	Recruiting (NCT05106504)	Unknown	2021–2024
Meganatural-Az grapeseed extract	AD	Active, not recruiting (NCT02033941)	Phase 2	2014–2021
Memantine and sodium oligomannate (GV-971)	AD	Not yet recruiting (NCT05430867)	Phase 4	2022–2024
Omega-3	AD	Recruiting (NCT03691519)	Phase 3	2018–2023
Omega 3 PUFA	AD	Active, not recruiting (NCT01953705)	Phase 2	2014–2021
Rapamycin	AD	Recruiting (NCT04629495)	Phase 2	2021–2024
Salsalate	AD	Active, not recruiting (NCT03277573)	Phase 1	2017–2021
Scopolamine, atropine, edaravone and dexmedetomidine	ALS	Not yet recruiting (NCT04391361)	Phase 2	2020–2023
SLS-005 (Trehalose injection)	AD	Not yet recruiting (NCT05332678)	Phase 2	2022–2024
Sodium oligomannate capsules (GV-971)	AD	Recruiting (NCT05058040)	Phase 4	2021–2024
Sodium oligomannate (GV-971)	AD	Recruiting (NCT04520412)	Phase 3	2020–2026
Sodium oligomannate capsules (GV-971)	AD	Recruiting (NCT05181475)	Phase 4	2021–2025
Sulforaphane	PD	Not yet recruiting (NCT05084365)	Phase 2	2021–2022
Trehalose	AD	Recruiting (NCT04663854)	Phase 1	2020–2022
Trehalose	PD	Not yet recruiting (NCT05355064)	Phase 4	2022–2023
Yangxue Qingnao pills	AD	Recruiting (NCT04780399)	Phase 2	2021–2024

Date: Estimated start date to estimated completion date.

## Data Availability

Not applicable.

## References

[1] Fu H., Hardy J., Duff K.E. (2018). Selective vulnerability in neurodegenerative diseases. Nat. Neurosci..

[2] Sun Y., Xia X., Basnet D., Zheng J.C., Huang J., Liu J. (2022). Mechanisms of ferroptosis and emerging links to the pathology of neurodegenerative diseases. Front. Aging Neurosci..

[3] Cunnane S.C., Trushina E., Morland C., Prigione A., Casadesus G., Andrews Z.B., Beal M.F., Bergersen L.H., Brinton R.D., de la Monte S. (2020). Brain energy rescue: An emerging therapeutic concept for neurodegenerative disorders of ageing. Nat. Rev. Drug Discov..

[4] Bastien J., Menon S., Messa M., Nyfeler B. (2021). Molecular targets and approaches to restore autophagy and lysosomal capacity in neurodegenerative disorders. Mol. Asp. Med..

[5] Esmaeili Y., Yarjanli Z., Pakniya F., Bidram E., Los M.J., Eshraghi M., Klionsky D.J., Ghavami S., Zarrabi A. (2022). Targeting autophagy, oxidative stress, and ER stress for neurodegenerative disease treatment. J. Control. Release.

[6] Chidambaram S.B., Essa M.M., Rathipriya A.G., Bishir M., Ray B., Mahalakshmi A.M., Tousif A.H., Sakharkar M.K., Kashyap R.S., Friedland R.P. (2022). Gut dysbiosis, defective autophagy and altered immune responses in neurodegenerative diseases: Tales of a vicious cycle. Pharmacol. Ther..

[7] Rao T., Tan Z., Peng J., Guo Y., Chen Y., Zhou H., Ouyang D. (2019). The pharmacogenetics of natural products: A pharmacokinetic and pharmacodynamic perspective. Pharmacol. Res..

[8] Su X., Zhou M., Li Y., Zhang J., An N., Yang F., Zhang G., Yuan C., Chen H., Wu H. (2022). Protective effects of natural products against myocardial ischemia/reperfusion: Mitochondria-targeted therapeutics. Biomed. Pharmacother..

[9] Dong S., Guo X., Han F., He Z., Wang Y. (2022). Emerging role of natural products in cancer immunotherapy. Acta Pharm. Sin. B.

[10] Rahman M.H., Bajgai J., Fadriquela A., Sharma S., Trinh T.T., Akter R., Jeong Y.J., Goh S.H., Kim C.S., Lee K.J. (2021). Therapeutic potential of natural products in treating neurodegenerative disorders and their future prospects and challenges. Molecules.

[11] Ciccone L., Vandooren J., Nencetti S., Orlandini E. (2021). Natural marine and terrestrial compounds as modulators of matrix metalloproteinases-2 (MMP-2) and MMP-9 in Alzheimer’s disease. Pharmaceuticals.

[12] Malar D.S., Prasanth M.I., Brimson J.M., Sharika R., Sivamaruthi B.S., Chaiyasut C., Tencomnao T. (2020). Neuroprotective properties of green tea (Camellia sinensis) in Parkinson’s disease: A review. Molecules.

[13] Sharifi-Rad M., Lankatillake C., Dias D.A., Docea A.O., Mahomoodally M.F., Lobine D., Chazot P.L., Kurt B., Tumer T.B., Moreira A.C. (2020). Impact of natural compounds on neurodegenerative disorders: From preclinical to pharmacotherapeutics. J. Clin. Med..

[14] Ye C., Liang Y., Chen Y., Xiong Y., She Y., Zhong X., Chen H., Huang M. (2021). Berberine improves cognitive impairment by simultaneously impacting cerebral blood flow and beta-amyloid accumulation in an APP/tau/PS1 mouse model of Alzheimer’s disease. Cells.

[15] Sharma N., Nehru B. (2018). Curcumin affords neuroprotection and inhibits alpha-synuclein aggregation in lipopolysaccharide-induced Parkinson’s disease model. Inflammopharmacology.

[16] Oliveira A.M., Cardoso S.M., Ribeiro M., Seixas R.S., Silva A.M., Rego A.C. (2015). Protective effects of 3-alkyl luteolin derivatives are mediated by Nrf2 transcriptional activity and decreased oxidative stress in Huntington’s disease mouse striatal cells. Neurochem. Int..

[17] Cai M., Yang E.J. (2016). Ginsenoside Re attenuates neuroinflammation in a symptomatic ALS animal model. Am. J. Chin. Med..

[18] Moren C., deSouza R.M., Giraldo D.M., Uff C. (2022). Antioxidant therapeutic strategies in neurodegenerative diseases. Int. J. Mol. Sci..

[19] Du X.X., Xu H.M., Jiang H., Song N., Wang J., Xie J.X. (2012). Curcumin protects nigral dopaminergic neurons by iron-chelation in the 6-hydroxydopamine rat model of Parkinson’s disease. Neurosci. Bull..

[20] Shao L., Dong C., Geng D., He Q., Shi Y. (2021). Ginkgolide B protects against cognitive impairment in senescence-accelerated P8 mice by mitigating oxidative stress, inflammation and ferroptosis. Biochem. Biophys. Res. Commun..

[21] Yang S., Xie Z., Pei T., Zeng Y., Xiong Q., Wei H., Wang Y., Cheng W. (2022). Salidroside attenuates neuronal ferroptosis by activating the Nrf2/HO1 signaling pathway in Abeta1-42-induced Alzheimer’s disease mice and glutamate-injured HT22 cells. Chin. Med..

[22] Gunesch S., Hoffmann M., Kiermeier C., Fischer W., Pinto A.F.M., Maurice T., Maher P., Decker M. (2020). 7-O-Esters of taxifolin with pronounced and overadditive effects in neuroprotection, anti-neuroinflammation, and amelioration of short-term memory impairment in vivo. Redox. Biol..

[23] Wang B., Zhong Y., Gao C., Li J. (2017). Myricetin ameliorates scopolamine-induced memory impairment in mice via inhibiting acetylcholinesterase and down-regulating brain iron. Biochem. Biophys. Res. Commun..

[24] Camandola S., Mattson M.P. (2017). Brain metabolism in health, aging, and neurodegeneration. EMBO J..

[25] Olzmann J.A., Carvalho P. (2019). Dynamics and functions of lipid droplets. Nat. Rev. Mol. Cell Biol..

[26] Castellanos D.B., Martin-Jimenez C.A., Rojas-Rodriguez F., Barreto G.E., Gonzalez J. (2021). Brain lipidomics as a rising field in neurodegenerative contexts: Perspectives with Machine Learning approaches. Front. Neuroendocrinol..

[27] Yin F. (2022). Lipid metabolism and Alzheimer’s disease: Clinical evidence, mechanistic link and therapeutic promise. FEBS J..

[28] Wang P., Su C., Feng H., Chen X., Dong Y., Rao Y., Ren Y., Yang J., Shi J., Tian J. (2017). Curcumin regulates insulin pathways and glucose metabolism in the brains of APPswe/PS1dE9 mice. Int. J. Immunopathol. Pharmacol..

[29] Li H., Jia J., Wang W., Hou T., Tian Y., Wu Q., Xu L., Wei Y., Wang X. (2018). Honokiol alleviates cognitive deficits of Alzheimer’s disease (PS1V97L) transgenic mice by activating mitochondrial SIRT3. J. Alzheimers. Dis..

[30] Zhang S., Wang S., Shi X., Feng X. (2020). Polydatin alleviates parkinsonism in MPTP-model mice by enhancing glycolysis in dopaminergic neurons. Neurochem. Int..

[31] Lu W.T., Sun S.Q., Li Y., Xu S.Y., Gan S.W., Xu J., Qiu G.P., Zhuo F., Huang S.Q., Jiang X.L. (2019). Curcumin ameliorates memory deficits by enhancing lactate content and MCT2 expression in APP/PS1 transgenic mouse model of Alzheimer’s disease. Anat. Rec..

[32] Wang J., Cui Y., Yu Z., Wang W., Cheng X., Ji W., Guo S., Zhou Q., Wu N., Chen Y. (2019). Brain endothelial cells maintain lactate homeostasis and control adult hippocampal neurogenesis. Cell Stem Cell.

[33] Chen J., Gao L., Zhang Y., Su Y., Kong Z., Wang D., Yan M. (2021). Acteoside-improved streptozotocin-induced learning and memory impairment by upregulating hippocampal insulin, glucose transport, and energy metabolism. Phytother. Res..

[34] Cisternas P., Oliva C.A., Torres V.I., Barrera D.P., Inestrosa N.C. (2019). Presymptomatic treatment with andrographolide improves brain metabolic markers and cognitive behavior in a model of early-onset Alzheimer’s disease. Front. Cell Neurosci..

[35] Zhang Y., Yang X., Wang S., Song S. (2019). Ginsenoside Rg3 prevents cognitive impairment by improving mitochondrial dysfunction in the rat model of Alzheimer’s disease. J. Agric. Food Chem..

[36] Zhang W., Xu C., Sun J., Shen H.M., Wang J., Yang C. (2022). Impairment of the autophagy-lysosomal pathway in Alzheimer’s diseases: Pathogenic mechanisms and therapeutic potential. Acta Pharm. Sin. B.

[37] Bonam S.R., Tranchant C., Muller S. (2021). Autophagy-lysosomal pathway as potential therapeutic target in Parkinson’s disease. Cells.

[38] Croce K.R., Yamamoto A. (2019). A role for autophagy in Huntington’s disease. Neurobiol. Dis..

[39] Amin A., Perera N.D., Beart P.M., Turner B.J., Shabanpoor F. (2020). Amyotrophic lateral sclerosis and autophagy: Dysfunction and therapeutic targeting. Cells.

[40] Jiang W., Wei W., Gaertig M.A., Li S., Li X.J. (2015). Therapeutic effect of berberine on Huntington’s disease transgenic mouse model. PLoS ONE.

[41] Huang M., Jiang X., Liang Y., Liu Q., Chen S., Guo Y. (2017). Berberine improves cognitive impairment by promoting autophagic clearance and inhibiting production of beta-amyloid in APP/tau/PS1 mouse model of Alzheimer’s disease. Exp. Gerontol..

[42] Chen Y., Chen Y., Liang Y., Chen H., Ji X., Huang M. (2020). Berberine mitigates cognitive decline in an Alzheimer’s disease mouse model by targeting both tau hyperphosphorylation and autophagic clearance. Biomed. Pharmacother..

[43] Deng H., Ma Z. (2021). Protective effects of berberine against MPTP-induced dopaminergic neuron injury through promoting autophagy in mice. Food Funct..

[44] Mancuso R., del Valle J., Modol L., Martinez A., Granado-Serrano A.B., Ramirez-Nunez O., Pallas M., Portero-Otin M., Osta R., Navarro X. (2014). Resveratrol improves motoneuron function and extends survival in SOD1(G93A) ALS mice. Neurotherapeutics.

[45] Zhang L., Park J.Y., Zhao D., Kwon H.C., Yang H.O. (2021). Neuroprotective effect of Astersaponin I against Parkinson’s disease through autophagy induction. Biomol. Ther..

[46] Gao L., Li X., Meng S., Ma T., Wan L., Xu S. (2020). Chlorogenic acid alleviates Abeta25-35-induced autophagy and cognitive impairment via the mTOR/TFEB signaling pathway. Drug Des. Devel. Ther..

[47] Chandra S., Roy A., Jana M., Pahan K. (2019). Cinnamic acid activates PPARalpha to stimulate lysosomal biogenesis and lower Amyloid plaque pathology in an Alzheimer’s disease mouse model. Neurobiol. Dis..

[48] Song J.X., Malampati S., Zeng Y., Durairajan S.S.K., Yang C.B., Tong B.C., Iyaswamy A., Shang W.B., Sreenivasmurthy S.G., Zhu Z. (2020). A small molecule transcription factor EB activator ameliorates beta-amyloid precursor protein and Tau pathology in Alzheimer’s disease models. Aging Cell.

[49] Long T., Wu Q., Wei J., Tang Y., He Y.N., He C.L., Chen X., Yu L., Yu C.L., Law B.Y. (2022). Ferulic acid exerts neuroprotective effects via autophagy induction in C. elegans and cellular models of Parkinson’s disease. Oxid. Med. Cell. Longev..

[50] Choy K.W., Murugan D., Mustafa M.R. (2018). Natural products targeting ER stress pathway for the treatment of cardiovascular diseases. Pharmacol. Res..

[51] Li J., Yu H., Yang C., Ma T., Dai Y. (2022). Therapeutic potential and molecular mechanisms of echinacoside in neurodegenerative diseases. Front. Pharmacol..

[52] Zhang Y., Long H., Zhou F., Zhu W., Ruan J., Zhao Y., Lu Y. (2017). Echinacoside’s nigrostriatal dopaminergic protection against 6-OHDA-Induced endoplasmic reticulum stress through reducing the accumulation of Seipin. J. Cell Mol. Med..

[53] Dai Y., Han G., Xu S., Yuan Y., Zhao C., Ma T. (2020). Echinacoside suppresses amyloidogenesis and modulates F-actin remodeling by targeting the ER stress sensor PERK in a mouse model of Alzheimer’s disease. Front. Cell Dev. Biol..

[54] Ge B., Li S.L., Li F.R. (2020). Astragaloside-IV regulates endoplasmic reticulum stress-mediated neuronal apoptosis in a murine model of Parkinson’s disease via the lincRNA-p21/CHOP pathway. Exp. Mol. Pathol..

[55] Liang Y., Ye C., Chen Y., Chen Y., Diao S., Huang M. (2021). Berberine improves behavioral and cognitive deficits in a mouse model of Alzheimer’s disease via regulation of beta-amyloid production and endoplasmic reticulum stress. ACS Chem. Neurosci..

[56] Wu Y., Chen Q., Wen B., Wu N., He B., Chen J. (2021). Berberine Reduces Abeta42 Deposition and Tau Hyperphosphorylation via Ameliorating Endoplasmic Reticulum Stress. Front. Pharmacol..

[57] Kou J., Wang M., Shi J., Zhang H., Pu X., Song S., Yang C., Yan Y., Doring Y., Xie X. (2021). Curcumin reduces cognitive deficits by inhibiting neuroinflammation through the endoplasmic reticulum stress pathway in apolipoprotein E4 transgenic mice. ACS Omega.

[58] Du K., Liu M., Zhong X., Yao W., Xiao Q., Wen Q., Yang B., Wei M. (2018). Epigallocatechin gallate reduces amyloid beta-induced neurotoxicity via inhibiting endoplasmic reticulum stress-mediated apoptosis. Mol. Nutr. Food Res..

[59] Tana, Nakagawa T. (2022). Luteolin ameliorates depression-like behaviors by suppressing ER stress in a mouse model of Alzheimer’s disease. Biochem. Biophys. Res. Commun..

[60] Chen J., Deng X., Liu N., Li M., Liu B., Fu Q., Qu R., Ma S. (2016). Quercetin attenuates tau hyperphosphorylation and improves cognitive disorder via suppression of ER stress in a manner dependent on AMPK pathway. J. Funct. Foods.

[61] Gaballah H.H., Zakaria S.S., Elbatsh M.M., Tahoon N.M. (2016). Modulatory effects of resveratrol on endoplasmic reticulum stress-associated apoptosis and oxido-inflammatory markers in a rat model of rotenone-induced Parkinson’s disease. Chem. Biol. Interact..

[62] He Y., Ruganzu J.B., Lin C., Ding B., Zheng Q., Wu X., Ma R., Liu Q., Wang Y., Jin H. (2020). Tanshinone IIA ameliorates cognitive deficits by inhibiting endoplasmic reticulum stress-induced apoptosis in APP/PS1 transgenic mice. Neurochem. Int..

[63] Sivamaruthi B.S., Suganthy N., Kesika P., Chaiyasut C. (2020). The role of microbiome, dietary supplements, and probiotics in Autism Spectrum Disorder. Int. J. Environ. Res. Public Health.

[64] Wang Q., Luo Y., Ray Chaudhuri K., Reynolds R., Tan E.K., Pettersson S. (2021). The role of gut dysbiosis in Parkinson’s disease: Mechanistic insights and therapeutic options. Brain.

[65] Shen L., Liu L., Li X.Y., Ji H.F. (2019). Regulation of gut microbiota in Alzheimer’s disease mice by silibinin and silymarin and their pharmacological implications. Appl. Microbiol. Biotechnol..

[66] Xie Z., Lu H., Yang S., Zeng Y., Li W., Wang L., Luo G., Fang F., Zeng T., Cheng W. (2020). Salidroside attenuates cognitive dysfunction in senescence-accelerated mouse prone 8 (SAMP8) mice and modulates inflammation of the gut-brain axis. Front. Pharmacol..

[67] Duan J., Pan J., Sun M., Fang Y. (2022). Comparative multiomics study of the effects of Ellagic acid on the gut environment in young and adult mice. Food Res. Int..

[68] Garcia-Villalba R., Gimenez-Bastida J.A., Cortes-Martin A., Avila-Galvez M.A., Tomas-Barberan F.A., Selma M.V., Espin J.C., Gonzalez-Sarrias A. (2022). Urolithins: A comprehensive update on their metabolism, bioactivity, and associated gut microbiota. Mol. Nutr. Food Res..

[69] Li J., Meng P., Zhang J., He M. (2022). Effect of berberine hydrochloride on the diversity of intestinal flora in Parkinson’s disease patients. Contrast Media Mol. Imaging.

[70] Sun Z.Z., Li X.Y., Wang S., Shen L., Ji H.F. (2020). Bidirectional interactions between curcumin and gut microbiota in transgenic mice with Alzheimer’s disease. Appl. Microbiol. Biotechnol..

[71] Fasina O.B., Wang J., Mo J., Osada H., Ohno H., Pan W., Xiang L., Qi J. (2022). Gastrodin from gastrodia elata enhances cognitive function and neuroprotection of AD mice via the regulation of gut microbiota composition and inhibition of neuron inflammation. Front. Pharmacol..

[72] Liu J., Ye T., Zhang Y., Zhang R., Kong Y., Zhang Y., Sun J. (2021). Protective effect of Ginkgolide B against cognitive impairment in mice via regulation of gut microbiota. J. Agric. Food Chem..

[73] Wang L., Lu J., Zeng Y., Guo Y., Wu C., Zhao H., Zheng H., Jiao J. (2020). Improving Alzheimer’s disease by altering gut microbiota in tree shrews with ginsenoside Rg1. FEMS Microbiol. Lett..

[74] Qu C., Li Q.P., Su Z.R., Ip S.P., Yuan Q.J., Xie Y.L., Xu Q.Q., Yang W., Huang Y.F., Xian Y.F. (2022). Nano-Honokiol ameliorates the cognitive deficits in TgCRND8 mice of Alzheimer’s disease via inhibiting neuropathology and modulating gut microbiota. J. Adv. Res..

[75] Li C., Wang N., Zheng G., Yang L. (2021). Oral administration of resveratrol-selenium-peptide nanocomposites alleviates Alzheimer’s disease-like pathogenesis by inhibiting Abeta aggregation and regulating gut microbiota. ACS Appl. Mater. Interfaces.

[76] Liu Y., Gong Y., Xie W., Huang A., Yuan X., Zhou H., Zhu X., Chen X., Liu J., Liu J. (2020). Microbubbles in combination with focused ultrasound for the delivery of quercetin-modified sulfur nanoparticles through the blood brain barrier into the brain parenchyma and relief of endoplasmic reticulum stress to treat Alzheimer’s disease. Nanoscale.

[77] Crowe T.P., Hsu W.H. (2022). Evaluation of recent intranasal drug delivery systems to the central nervous system. Pharmaceutics.

[78] Lofts A., Abu-Hijleh F., Rigg N., Mishra R.K., Hoare T. (2022). Using the intranasal route to administer drugs to treat neurological and psychiatric illnesses: Rationale, successes, and future needs. CNS Drugs.

[79] Dou Y., Zhao D., Yang F., Tang Y., Chang J. (2021). Natural phyto-antioxidant albumin nanoagents to treat advanced Alzheimer’s disease. ACS Appl. Mater. Interfaces.

[80] Liu J., Liu C., Zhang J., Zhang Y., Liu K., Song J.X., Sreenivasmurthy S.G., Wang Z., Shi Y., Chu C. (2020). A self-assembled alpha-synuclein nanoscavenger for Parkinson’s disease. ACS Nano.

